# A comparison of the prognostic value of BNP versus NT-proBNP after hospitalisation for heart failure

**DOI:** 10.1007/s12471-018-1145-x

**Published:** 2018-08-07

**Authors:** G. C. M. Linssen, T. Jaarsma, H. L. Hillege, A. A. Voors, D. J. van Veldhuisen

**Affiliations:** 1 Department of Cardiology, University Medical Center Groningen, University of Groningen, Groningen, The Netherlands; 2 Department of Cardiology, Hospital Group Twente, Almelo and Hengelo, The Netherlands; 3 Department of Social and Welfare Studies, Faculty of Health Sciences, Linköping University, Norrköping, Sweden; 4 Department of Epidemiology, University Medical Center Groningen, University of Groningen, Groningen, The Netherlands

**Keywords:** Heart failure, Biomarkers, B-type natriuretic peptides, BNP, NT-proBNP, Prognosis

## Abstract

**Aims:**

Concentrations of circulating B‑type natriuretic peptides provide important prognostic information in heart failure (HF) patients. We directly compared the prognostic performance of brain natriuretic peptide (BNP) versus N‑terminal-proBNP (NT-proBNP) measurements in a large population of HF patients at hospital discharge after an admission for decompensated HF.

**Methods and results:**

BNP and NT-proBNP were measured in 563 stable HF patients before discharge. All patients were followed for a fixed period of 18 months. The primary endpoint was time to first major event (HF hospitalisation or death).

Patients were in NYHA class II (47%) or III/IV (53%) at discharge and the mean age of the patients was 71 ± 11 years, 217 (39%) females, mean left ventricular ejection fraction was 0.32 ± 0.14 and 234 (42%) had an ischaemic aetiology of HF. During the study, 236 patients (42%) reached the primary endpoint. Multivariate odds ratios of the primary endpoint for doubling of baseline levels of BNP and NT-proBNP were 1.46 (95% CI 1.19–1.80, *p* < 0.001) and 1.45 (95% CI 1.18–1.78, *p* < 0.001), respectively. The multivariable adjusted areas under the receiver-operating characteristic curve for prediction of the primary endpoint for doubling of BNP and NT-proBNP were 0.69 and 0.68, respectively. Direct comparison of the prognostic value of BNP and NT-proBNP did not reveal significant differences.

**Conclusions:**

BNP and NT-proBNP at discharge for hospitalisation for HF are powerful, and equally strong and independent predictors of all-cause death and HF rehospitalisation.

## What’s new?

- In patients with decompensated heart failure (HF), both B‑type natriuretic peptides (BNP and NT-proBNP) are equally strong and independent predictors of outcome (HF rehospitalisation or death)
- Both natriuretic peptides have comparable predictive accuracy at hospital discharge for HF.
- Clinically relevant for disease monitoring in HF patients treated with exogenous BNP or sacubitril/valsartan.
- Important for the selection of patients in trials and usage of natriuretic peptides as endpoints.

## Introduction

Hospitalisation for acute heart failure (HF) syndromes portends a poor prognosis in patients with chronic HF [1]. Brain natriuretic peptides (BNP) and its equimolarly secreted amino-terminal fragment (NT-proBNP) are strong independent predictors of mortality and cardiovascular (CV) events in patients with heart failure (HF) [2–4]. Therefore, these biomarkers can help to identify patients at high risk for premature death or HF (re)hospitalisation.

Although both natriuretic peptides are frequently used, direct comparative studies on the prognostic value of BNP and NT-proBNP are scarce. Since BNP and NT-proBNP differ with regard to their biological activity and half-life, in vitro stability and clearance mechanisms, potential differences regarding their prognostic value may exist. In the Val-HeFT study the prognostic values of BNP and NT-proBNP were compared in patients with stable chronic HF and in that study NT-proBNP was better in terms of predicting outcome [5]. However, predicting outcome after hospital discharge for worsening HF may be more difficult, but is clinically relevant. In this condition, only the findings from relatively small comparative studies are available [6–8].

Therefore, the purpose of our present study was to directly compare the prognostic performance of BNP versus NT-pro-BNP measurements in a large population of HF patients at hospital discharge after an admission for decompensated HF.

## Methods

### Patient population and study design

This analysis was performed as part of the Coordinating study evaluating Outcomes of Advising and Counselling in Heart Failure (COACH) study, a multicentre, randomised, open trial with blinded endpoint evaluation, in which 1,023 patients were enrolled. It was designed to compare basic support and intensive support in patients with chronic HF to a control group receiving ‘usual’ care, as described in detail before [9]. All patients had been admitted to hospital with symptoms of HF, New York Heart Association (NYHA) functional class II–IV. Patients were ±18 years of age and had evidence of structural underlying heart disease, as shown by cardiovascular imaging. Both patients with an impaired and those with a preserved left ventricular ejection fraction could participate. Before discharge from the hospital (i. e. before inclusion into the study), patients had to be stable on standard medication for HF, at the discretion of the physician and if tolerated. After inclusion, all patients were followed for a fixed time period of 18 months. Primary endpoints were time to death or rehospitalisation for HF, and the number of days lost to death or hospitalisation. The Medical Ethics Committee approved the study protocol and all patients provided written informed consent. Institutional review board approval was given for all 17 participating Dutch centres. The primary results of the COACH study have been published previously [10].

In the current analyses, we only investigated COACH patients in whom measurements of plasma levels of both BNP and NT-proBNP at hospital discharge for decompensated chronic heart failure (baseline) were available. Of the 1,023 patients included in the COACH study, 563 patients had both BNP and NT-proBNP plasma levels available. The main reason for missing BNP and NT-proBNP data was temporary unavailability of the necessary laboratory facilities, usually in the starting phase of the study. Details have been published previously [11]. We studied the primary endpoint, time to first major event (HF hospitalisation or death).

Previously we reported specifically on the prognostic significance of BNP (and NT-proBNP) in heart failure patients with reduced and preserved left ventricular ejection fraction (LVEF) (≤40% and >40%, respectively) who participated in the COACH study [3]. In the current analyses we introduced LVEF as a continuous variable.

### Statistical analyses

Continuous variables with a normal distribution are expressed as means with standard deviation (SD). Nominal variables are expressed as *n* (%). Levels of BNP and NT-proBNP with a skewed distribution are given as medians with interquartile range (IQR). Differences in continuous variables were evaluated by Student’s *t*‑test or Mann-Whitney-U tests, depending on normality of data. Categorical clinical variables were compared with the Fisher’s exact test or chi-square test. To achieve a constant variance, natriuretic peptide values were logarithmically transformed. BNP levels were correlated with NT-proBNP levels using Spearman’s rank correlation coefficient.

The primary endpoint was time to first major event (HF hospitalisation or death). To estimate the size of the effect, odds ratios (ORs) with 95% confidence intervals (CIs) were calculated with the use of logistic regression models. A stepwise approach was used.

Risk estimates should be interpreted as the relative risk if values of BNP or NT-proBNP were doubled (e. g., from 10 to 20 pg/ml). From logistic regression analysis the predictive values of BNP and NT-proBNP were determined and the area under the receiver operating characteristics (ROC) curves (AUC) for quantification of the predictive accuracy were calculated. An ROC area of 0.5 signifies no discriminatory value, while an area of 1.0 means perfect discrimination for prediction of those with and without an endpoint during follow-up.

Furthermore, a direct comparison of single levels in the regression models in COACH patients was part of this analysis.

All reported probability values are two-tailed and *p* < 0.05 was considered statistically significant. Analyses were performed using STATA software (STATA version 10.0, College Station, Texas, USA).

## Results

### Baseline characteristics and outcome

The study group at baseline comprised 563 HF patients: 217 (39%) females, the mean age of the patients was 71 ± 11 years. Patients were in NYHA class II (47%) or III/IV (53%) at discharge. Both in the total cohort of COACH (*n* = 1,023) and in our subgroup (*n* = 563), only 4% of HF patients were in NYHA class IV at hospital discharge. Therefore, we clustered NYHA class III and IV in the current analyses.

The mean LVEF was 0.32 ± 0.14 and 234 (42%) had an ischaemic aetiology of HF. Mean estimated glomerular filtration rate (eGFR) was 54 ± 20 ml/min/1.73 m^2^. There were no significant differences in baseline characteristics between patients who participated in this study and in the main COACH cohort. In Tab. 1 baseline characteristics of the study group (*n* = 563) at hospital discharge are presented.

**Table 1:** Baseline characteristics of the study group at hospital discharge

	Study group (*n* = 563)
Female gender	217 (39%)
Age (years)	71 ± 11
NYHA class III or IV^a^	294 (53%)
LVEF	0.32 ± 0.14
Body mass index (kg/m^2^)	26 ± 5
Aetiology of heart failure	
– Ischaemic heart disease	234 (42%)
– Non-ischaemic heart disease	329 (58%)
Previously hospitalised for HF	192 (34%)
Comorbidities	
– Hypertension	249 (44%)
– Atrial fibrillation	258 (46%)
– Diabetes	167 (30%)
– Stroke	51 (9%)
– COPD	156 (28%)
Medication	
– ACE-I and/or ARB	463 (82%)
– β-blockers	375 (67%)
– Diuretics^b^	538 (96%)
Laboratory values	
– Haemoglobin (mmol/l)	8.2 ± 1.3
– eGFR (ml/min/1.73 m^2^)	54 ± 20
– BNP (pg/ml)^c^	447 (196–906)
– NT-proBNP (pg/ml)^c^	2,528 (1289–5615)

SI conversion factors: To convert NT-proBNP to picomoles per litre, divide by 8.46; BNP to picomoles per litre, divide by 3.47; haemoglobin to grams per litre, divide by 0.62

All continuous variables are presented as mean ± SD

*ACE-I* angiotensin-converting enzyme inhibitor, *ARB* angiotensin receptor blocker, *BNP* brain natriuretic peptide, *COPD* chronic obstructive pulmonary disease, *eGFR* estimated glomerular filtration rate, *HF* heart failure, *LVEF* left ventricular ejection fraction, *NT-proBNP* N-terminal prohormone B‑type natriuretic peptide, *NYHA* New York Heart Association

^a^NYHA class at hospital discharge

^b^Includes loop diuretics, thiazides, and aldosterone antagonists

^c^A continuous variable is presented as median value (25^th^–75^th^ percentiles)

During the 18-month study, the combined endpoint occurred in 236 patients (42%) of the whole population.

### BNP, NT-proBNP and outcome

At hospital discharge after an admission for decompensated HF, median (25^th^–75^th^ percentiles) BNP and NT-proBNP levels in the 563 study patients were 447 (196–906) and 2,528 (1,289–5,615) pg/ml, respectively (Tab. 1). We found a strong association between BNP and NT-proBNP concentrations (correlation coefficients 0.82, *p* < 0.001). BNP at baseline was significantly (*p* < 0.05) related to: NYHA class, LVEF, body mass index (BMI), and HF underlying disease (ischaemic vs. non-ischaemic). NT-proBNP at baseline was significantly related to age, NYHA class, LVEF, BMI, HF underlying disease (ischaemic vs. non-ischaemic), haemoglobin and eGFR.

The baseline median BNP and NT-proBNP levels were higher in patients (*n* = 236, 42%) who reached the primary endpoint compared with those (*n* = 337, 58%) who remained free of this endpoint: 559 vs. 352 pg/ml, *p* < 0.001; and 3,396 vs. 2,011 pg/ml, *p* < 0.001, respectively.

**Fig. 1:**
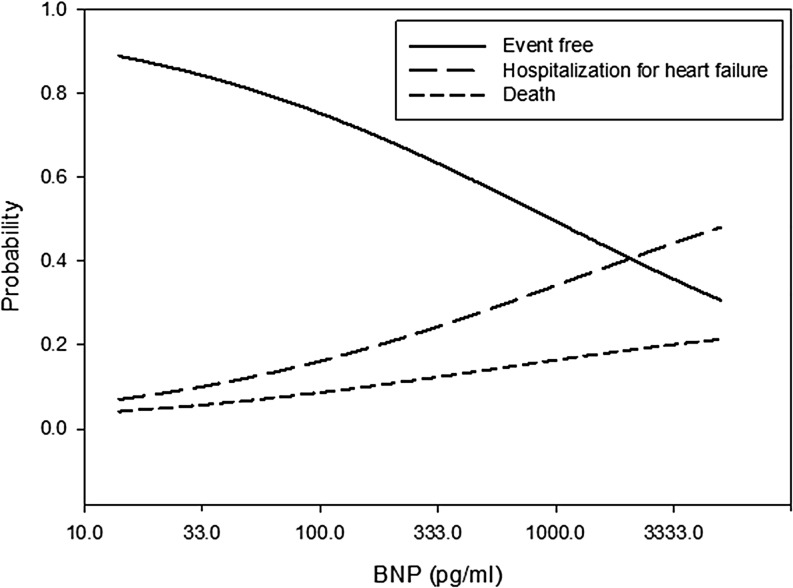
Multivariable adjusted probability of outcome according to the BNP level at hospital discharge (on a log transformed scale)

**Fig. 2:**
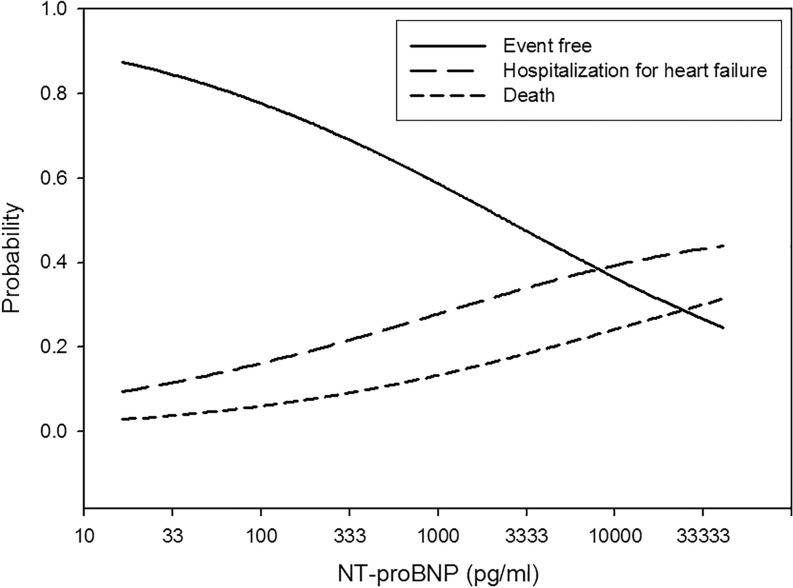
Multivariable adjusted probability of outcome according to the NT-proBNP level at hospital discharge (on a log transformed scale)

The multivariable adjusted probability for event-free survival and for the separate endpoints (HF hospitalisation and all-cause death) according to the baseline BNP and NT-proBNP levels, respectively, are depicted in Figs. 1 and 2.

### Prognostic value of a single predischarge measurement of BNP and NT-proBNP

Unadjusted, age- and gender-adjusted and multivariate ORs of the primary endpoint for doubling of baseline levels of BNP and NT-proBNP are presented in Tables 2 and 3.

**Table 2:** Odds ratios for outcome according to doubling of B‑type natriuretic peptide (BNP) at hospital discharge for heart failure

	Odds ratio	95% CI	*P* value
*For primary endpoint*			
Unadjusted model	1.30	1.16–1.45	<0.001
Model 1	1.29	1.15–1.45	<0.001
Model 2	1.46	1.19–1.80	<0.001
*For HF hospitalisation*			
Unadjusted model	1.24	1.09–1.41	<0.001
Model 1	1.25	1.09–1.41	<0.001
Model 2	1.42	1.20–1.68	<0.001
*For all-cause death*			
Unadjusted model	1.41	1.20–1.66	<0.001
Model 1	1.40	1.19–1.66	<0.001
Model 2	1.37	1.12–1.69	0.003

Model 1: Age- and gender-adjusted model

Model 2: Multivariate model adjusted for significant covariates: age, NYHA class, LVEF, BMI, HF underlying disease (ischaemic vs. non-ischaemic), and eGFR

**Table 3:** Odds ratios for outcome according to doubling of N‑terminal pro-B-type natriuretic peptide (NT-proBNP) at hospital discharge for heart failure

	Odds ratio	95% CI	*P* value
*For primary endpoint*			
Unadjusted model	1.34	1.20–1.49	<0.001
Model 1	1.31	1.17–1.46	<0.001
Model 2	1.45	1.18–1.78	<0.001
*For HF hospitalisation*			
Unadjusted model	1.23	1.10–1.40	<0.001
Model 1	1.22	1.08–1.38	<0.001
Model 2	1.33	1.13–1.56	<0.001
*For all-cause death*			
Unadjusted model	1.55	1.33–1.82	<0.001
Model 1	1.50	1.28–1.76	<0.001
Model 2	1.45	1.17–1.78	<0.001

Model 1: Age and gender adjusted model

Model 2: Multivariate model adjusted for significant covariates: age, NYHA class, LVEF, BMI, HF underlying disease (ischaemic vs. non-ischaemic), and eGFR

Multivariate ORs (adjusted for age, NYHA class, LVEF, BMI, HF underlying disease (ischaemic vs. non-ischaemic), and eGFR) were 1.46 (95% CI 1.19–1.80, *p* < 0.001) and 1.45 (95% CI 1.18–1.78, *p* < 0.001), respectively for BNP and NT-proBNP. In addition, both BNP and NT-proBNP were independent predictors of the separate endpoints of HF hospitalisation and all-cause death.

### Predictive accuracy of BNP versus NT-proBNP

The areas under the ROC curve for prediction of the primary endpoint, calculated for each doubling of BNP and NT-proBNP, were 0.69 and 0.68 respectively.

**Fig. 3:**
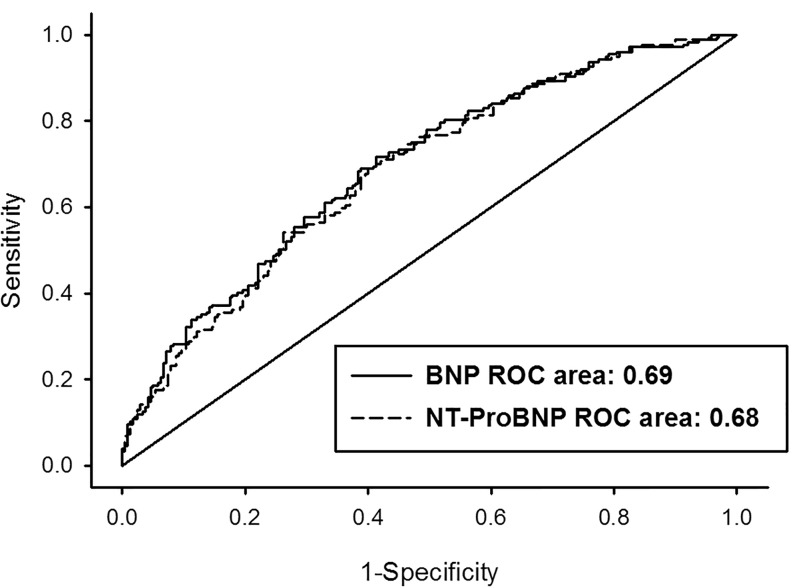
Receiver operating characteristics (ROC) curves (multivariable adjusted) of discharge levels of BNP and NT-proBNP levels in predicting hospitalisation for HF or death, both at 18 months

The corresponding, multivariable adjusted ROC curves for each doubling of BNP and NT-proBNP are plotted in Fig. 3. There were no significant differences in AUC between BNP and NT-proBNP.

## Discussion

The main finding of the present study is that BNP and NT-proBNP are equally strong and independent predictors of outcome at hospital discharge. Direct comparison of the predictive accuracy of BNP and NT-proBNP did not reveal significant differences.

Rational use of these peptides is currently recommended in patients with HF in several clinical settings: on admission for decompensated heart failure, after a major treatment effect, at hospital discharge when euvolaemia is reached, and during ambulatory follow-up [2, 12]. A single measurement of a B-type natriuretic peptide provides strong and independent prognostic information in patients with heart failure.

Previously, we observed in the COACH study that BNP levels are lower in patients with HF with preserved ejection fraction (HFpEF) than in patients with HF with reduced ejection fraction (HFrEF), but for a given BNP level, the prognosis in patients with HFpEF is as poor as in those with HFrEF [3]. Patients with HFpEF were older, more often female, and had a higher systolic blood pressure and body mass index, and haemoglobin levels were lower than in those with HFrEF.

Inclusion of BNP or NT-proBNP concentrations in the diagnostic algorithm of HF is important both in clinical decision making and for proper design of trials. In concordance with proposed pathophysiological concepts, our recent analyses of multiple biomarkers from COACH showed that in HFpEF patients, inflammation and angiogenesis-mediated interactions were predominant, while stretch-mediated interactions were found in HFrEF [2, 13–16].

Interestingly, in HFpEF patients with low BNP (<100 pg/ml) or low NT-proBNP (<300 pg/ml), quality of life, heart failure-related symptoms and clinical outcomes were similar to those with elevated BNP levels [17].

Furthermore, we reported on the added value of a diverse group of 29 biomarkers on top of a clinical risk model in COACH with and without NT-proBNP. Low risk was defined as a biomarker cut-off at the 10th percentile associated with high positive predictive value for 30-day and 180-day mortality and HF rehospitalisation [18].

HF symptom relief and euvolaemia reached at hospital discharge is an important point of time for measurement of BNP or NT-proBNP. Their concentrations may serve as targets for optimal fluid status or markers of disease evolution during follow-up in addition to clinical parameters, and in biomarker-guided management in HFrEF [19].

### BNP versus NT-proBNP in heart failure

Plasma levels of the biological active BNP and inactive N‑terminal fragment of BNP are closely correlated with each other in HF patients, as confirmed by the results of our study. The Valsartan Heart Failure Trial (Val-HeFT) study group provided a direct comparison of the prognostic value of BNP and NT-proBNP in 3,916 patients with chronic and stable HF [5]. They found that both peptides were powerful independent markers of outcome in HF, but NT-proBNP was superior to BNP in predicting mortality and morbidity or hospitalisation for HF. In 164 patients (99% men) hospitalised for decompensated HF, Waldo and coworkers found that admission and discharge NT-proBNP (AUC 0.788 and AUC 0.834) had superior prognostic power for all-cause mortality within 90 days post-discharge, when compared with BNP (AUC 0.644, *p* < 0.01 and AUC 0.709, *p* < 0.01) [6]. Also in a small study, 171 patients with acute HF, Noveanu and coworkers reported that predischarge levels of BNP and NT-proBNP reliably predicted one-year mortality (AUC 0.78 and 0.77 respectively); however, prediction of one-year HF readmission was poor for both markers [7]. In a subgroup analysis of 306 patients with acute HF (FINN-AKVA cohort), both BNP and NT-proBNP failed to improve prediction of 5‑year survival [8]. In 563 patients, we found that the prognostic performance (all-cause mortality or HF hospitalisation during 18 months) of BNP and NT-proBNP at the time of hospital discharge were comparable.

From other studies, it became apparent that patient- and assay-related factors influence both BNP and NT-proBNP concentrations [2, 20]. In head-to-head comparisons, distinct discrepancies in individual patients demonstrated that both markers are clinically not completely equivalent [7–9, 21, 22]. Furthermore, BNP was found to be more sensitive to rapid haemodynamic changes in acute heart failure than NT-proBNP [8, 23]. Importantly, monitoring by means of BNP testing of chronic heart failure patients on exogenous administered BNP or guideline-recommended sacubitril-valsartan treatment, may be impaired, in contrast to NT-proBNP, which is not a substrate for neprilysin inhibition [24]. So, these issues should be taken into account while applying serial testing for risk stratification and (long-term) monitoring of HF patients. Analyses of NT-proBNP and BNP in HFrEF patients who participated in the PARADIGM-HF study revealed that NT-proBNP decreases on treatment with sacubutril-valsartan, reflecting reduced cardiac wall stress, while BNP increases, reflecting drug action [25]. However, the relative increases in BNP concentrations in that randomised study were small (median baseline BNP value approximately 200 pg/ml to a median of about 225 pg/ml in the sacubitril-valsartan arm at 8‑month follow-up).

Furthermore, it is unlikely that small increments of BNP on that drug will interfere with the diagnostic applications of BNP in patients with acute or decompensated heart failure, usually associated with large increments of BNP.

### Study limitations

The present analysis was observational in design and is therefore only hypothesis-generating. In the current retrospective analysis of a randomised controlled trial, we only included medical therapy at hospital discharge. In that way, modifications in the drug treatment and non-pharmacological therapy during follow-up were not accounted for in our analysis. The COACH study was powered for the primary composite endpoint, time to hospitalisation for HF or all-cause mortality, but not for the separate, secondary endpoint all-cause mortality.

Also, the COACH study was conducted at a time when not all currently recommended drugs for chronic HF were used and use of device therapy has also markedly increased since then, which may have affected our findings [12, 26].

## Conclusions

Plasma concentrations of both BNP and NT-proBNP at discharge after hospitalisation for HF are equally strong and independent predictors of all-cause death and HF rehospitalisation.
